# Dynamics of Uptake and Metabolism of Small Molecules in Cellular Response Systems

**DOI:** 10.1371/journal.pone.0004923

**Published:** 2009-03-17

**Authors:** Maria Werner, Szabolcs Semsey, Kim Sneppen, Sandeep Krishna

**Affiliations:** 1 Department of Computational Biology, Royal Institute of Technology, Albanova University Center, Stockholm, Sweden; 2 Department of Genetics, Eotvos Lorand University, Budapest, Hungary; 3 Center for Models of Life, Niels Bohr Institute, University of Copenhagen, Copenhagen, Denmark; University of Arizona, United States of America

## Abstract

**Background:**

Proper cellular function requires uptake of small molecules from the environment. In response to changes in extracellular conditions cells alter the import and utilization of small molecules. For a wide variety of small molecules the cellular response is regulated by a network motif that combines two feedback loops, one which regulates the transport and the other which regulates the subsequent metabolism.

**Results:**

We analyze the dynamic behavior of two widespread but logically distinct two-loop motifs. These motifs differ in the logic of the feedback loop regulating the uptake of the small molecule. Our aim is to examine the qualitative features of the dynamics of these two classes of feedback motifs. We find that the negative feedback to transport is accompanied by overshoot in the intracellular amount of small molecules, whereas a positive feedback to transport removes overshoot by boosting the final steady state level. On the other hand, the negative feedback allows for a rapid initial response, whereas the positive feedback is slower. We also illustrate how the dynamical deficiencies of one feedback motif can be mitigated by an additional loop, while maintaining the original steady-state properties.

**Conclusions:**

Our analysis emphasizes the core of the regulation found in many motifs at the interface between the metabolic network and the environment of the cell. By simplifying the regulation into uptake and the first metabolic step, we provide a basis for elaborate studies of more realistic network structures. Particularly, this theoretical analysis predicts that FeS cluster formation plays an important role in the dynamics of iron homeostasis.

## Introduction

Cells are constantly assessing and responding to environmental changes, thereby maintaining a regulated system that deals with the presence of nutrients, minerals, and toxic compounds. To manipulate the many small molecules required for cellular function, cells regulate the uptake and the metabolism of these molecules. Typically, small molecules bind and alter the function of specific transcriptional regulators that regulate transcription of genes encoding the cognate transporters and metabolic enzymes [1]. In many cases the same transcription factor regulates both the transport and the metabolism genes, thus linking the two feedback loops [1], [2].

For instance, in *E. coli* the *lac* operon, repressed by LacI, contains genes for both lactose transport and metabolism [3]. Similarly, GalR, the main regulator of the galactose response system in *E. coli* controls both the D-galactose transporters and enzymes of galactose metabolism [4], [5]. Some metals (e.g. Fe, Zn, Cu) are necessary for living cells as components of important metalloproteins, but are detrimental at excessive concentrations [6], [7], [8]. In the best characterized iron regulatory network, in *E. coli*, the transcription factor Fur regulates the transcription of iron transport genes, as well as genes encoding iron-using proteins through regulating transcription of the small RNA RyhB [9]. In contrast, Zn import and export in *E. coli* is regulated by distinct regulators [10].

Another important facet of small molecular regulation is the fact that the turn around time for any small molecule is very short. Thus the timescale for changes in the intrinsic concentration of a small molecule is often orders of magnitude faster than the time needed to change the concentration of a typical protein. For example it is typically expected that the entire reservoir of lactose is reshuffled at least 1000 times in an *E. coli* generation [11], and the free Fe is replenished around 100 times [12]. This separation of timescales means that the consumption rate of the small molecule is much larger than the rate of dilution due to cell division, thereby making the dependence of its concentration on metabolism as important as its dependence on uptake.

**Figure 1:**
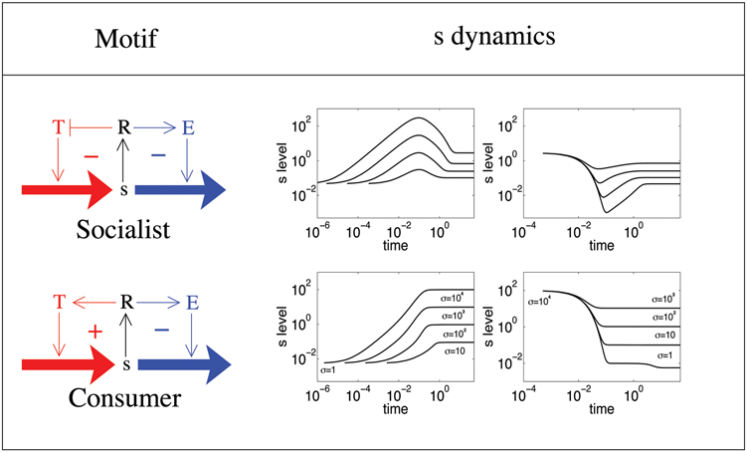
Dynamics of the socialist and consumer motifs. The left column of plots show the dynamics of intracellular *s* levels, as a function of time, for the corresponding motifs, for four different up-shifts in extracellular levels from *σ* to *σ*+Δ*σ* with Δ*σ* = 10, 10^2^, 10^3^ and 10^4^. The starting value is *σ* = 1. The right column shows similar dynamics of *s* for downshifts: Δ *σ* = −9000, −9900, −9990 and −9999. The starting value is *σ* = 10^4^. Time is measured in units of one cell generation.

We examine the qualitative dynamical behaviour of regulatory motifs consisting of two entangled feedback loops where a single regulator controls both the uptake (influx) and metabolism (out-flux) of a small molecule. The logically distinct combinations of two feedback loops, shown in Fig. 1, suit the requirements for regulation of different classes of small molecules, and are found, respectively, in networks regulating the response to necessary but potentially toxic molecules (e.g. Fe, Zn), and in different sugar networks (e.g. lactose, galactose). We call the motif found in metal ion regulation systems the *socialist* motif, and the one found in sugar response systems the *consumer* motif. The names originate from our previous study of the steady-state properties of these motifs [2]: the socialist motif is so named because it tightly constrains the steady-state intracellular small molecule concentration, while the consumer results in a sharp increase of transport and consumption when the small molecule is present extracellularly.

Both motifs have a tightly regulated set of proteins that exerts a negative feedback upon any increase in intrinsic level of the small molecule. The two motifs differ however in the logic of the feedback to transport. The *consumer* uses a positive feedback, in the sense that an increase in the intracellular concentration of small molecules increases the transport. The *socialist* uses negative feedback where an increase in intracellular levels shuts down transport. Here, we analyze the dynamical behavior of the motifs, in particular their response to small and large perturbations in the extracellular small molecule concentration. The socialist motif shows a very fast initial response but at the cost of producing a large overshoot in the intracellular small molecule concentration. The consumer does not produce an overshoot, but is relatively slow to respond if the perturbation is large enough to switch on or off the positive feedback.

Our analysis emphasizes the core of the regulation found in many motifs at the interface between the metabolic network and the environment of the cell. By simplifying the regulation into uptake and the first metabolic step, we provide a basis for elaborate studies of more realistic network structures, which in fact are often characterized by multiple feedbacks on both uptake and metabolic sides of the central regulator [5], [13]. We illustrate how one of these simple motifs can be seen as a core element of the iron regulation in *E. coli*, a complicated regulatory circuit which consists of a socialist motif enhanced by at least one additional positive feedback loop involving the use of iron to form FeS clusters [12], [14], [15]. Considering the results on the advantages and disadvantages of the socialist motif, this theoretical analysis predicts that FeS cluster formation plays an important role in the dynamics of iron homeostasis.

## Methods

In the two motifs, the dynamics of the intracellular level of small molecule *s*, transporter *T* and metabolism enzyme *E*, obey the equations
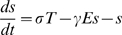
(1)

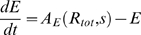
(2)

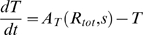
(3)The rate of transport of *s* into the cell, *σ*, is a measure of the extracellular level of the small molecule. The unit of time is chosen to be one cell generation. Thus, the rate of dilution of *s* due to cell growth is unity. We assume that this rate is much smaller than the metabolic consumption rate *γ*. In writing the transport and consumption rate as proportional to the extra- and intra-cellular small molecule concentrations, respectively, we are assuming that these concentrations are well below levels which would saturate the proteins. The metabolic enzymes and transport proteins are assumed to be very stable (as is the case for the galactose and Fe systems in *E. coli* [4], [12]), thus their concentration reduces only by dilution due to cell growth, therefore also has a rate equal to unity. Production of *E* and *T* are governed by the activity *A_E/T_*_._. *A_E/T_* describes the total rate of production, including transcription and translation, and depends on the interplay between the small molecule *s* and the regulator *R*. The small molecule *s* interacts with the regulator *R*, and this complex formation is assumed to be at equilibrium (i.e., the association and dissociation timescales are much faster than other timescales of the system). Depending on whether *s* inhibits or activates the regulator, the active form, R*, is either the free form *R_free_* or the complexed form *{Rs}*;
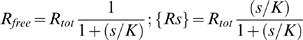
(4)where *R_tot_* is the total *R*, set as much smaller than *s*, and *K* is the dissociation constant for the *{Rs}* complex. All results discussed in the paper concern motifs where *s* activates *R*, However, the results are qualitatively similar for the motifs where *s* inhibits *R* (see supplementary material, Text S1). It is always possible to choose units of *s* such that *K* = 1. Therefore, without loss of generality we will henceforth set *K* = 1.

The active regulator in turn either activates or inhibits the production of *E* and *T*:
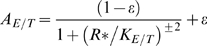
(5)where +2 is used when *R* represses production and −2 when *R* activates production. *ε* is the constitutive production rate and *K_E/T_* sets the binding strength of the *{RE}* or *{RT}* complex.

Default parameters are *R_to_*_t_ = 10, *K* = 1, *K_E/T_* = 1, *γ* = 100 and *ε* = 0.01. *γ* is chosen to be much larger than the dilution rate (unity). The exact value of *γ* does not affect our results qualitatively as long as *γ*≫1 (as mentioned in the Introduction, *γ* is typically of the order of 100 or more). *ε* is chosen to be small compared to the maximum rate of production (unity). Again, the exact value is unimportant as long as *ε*≪1. Further, we have chosen units of *T* and *E* such that their maximal production rates are unity. This is always possible, and does not lead to any loss of generality because the differences in production rate are absorbed into the parameters *σ* and *γ*. In choosing *K_E_* = *K_T_* we are exploring the behaviour of these motifs when both feedback loops are equal in strength (the qualitative dynamics we report is only changed if one of the loops is weakened so much as to be effectively absent). Given this, it is always possible to choose *K_E/T_* = 1 by an appropriate choice of units for measuring *R_tot_*. In these units, we have chosen *R_tot_* = 10 to allow around a 10-fold activation/repression of the transport and metabolic enzymes. This is in fact a conservative choice; repression of the *lac* operon by LacI, for example, is around 1000-fold [11]. Overall, we believe our choice of parameter values reasonably reflects typical biological systems with these feedback motifs.

## Results

We will denote the intracellular concentrations of the small molecule, transport proteins and metabolic enzymes by *s*, *T* and *E*, respectively. When the system is subject to perturbations in the extracellular small molecule concentration, *σ*, these intracellular concentrations change in time according to a set of ordinary differential equations described in the Methods section.

### Response to changes in extracellular small molecule level

Fig. 1 shows the time course of *s* in response to various up- and downshifts in *σ*, for both motifs. The difference in the steady state behavior of the two motifs stems from the fact that the socialist motif has more negative feedback than the consumer motif: As negative feedback is increased, it becomes increasingly difficult to import an excessively abundant resource, and its intrinsic levels therefore become self limiting for the socialist [2]. Thus, in all the plots of Fig. 1 the socialist shows a much narrower range of steady state *s* levels than the consumer, for the same range of *σ*.

**Figure 2:**
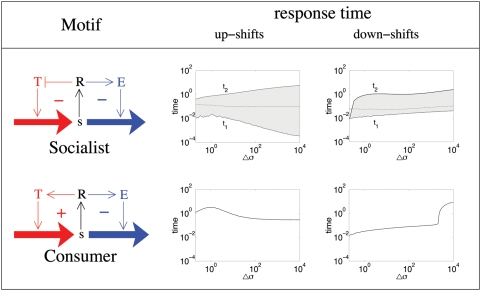
Response times of the socialist and consumer motifs. The plots show the response time of *s* as a function of the change in extra-cellular level, Δ*σ*, for both up- and downshifts in *σ*. The response time is defined as the time required to get to 95% of the final steady state levels of *s*. For the socialist motif there are two such response times, before (t_1_) and after (t_2_) the overshoot (solid lines). The socialist plots also shows the time of peak overshoot (dashed line) and the shaded region defines the duration of the overshoot.

The plots of Fig. 1 show a distinct difference between the socialist and the consumer in the shape of the curves: the socialist has a large overshoot in *s* levels for both up- and downshifts. Because of the overshoot, the socialist is relatively slow to reach its final steady state, taking around one cell generation (see also Fig. 2). This timescale comes directly from the timescale of degradation of *E* and *T* that we set in our equations to be one cell generation. On the other hand, in systems where the initial response is important and not the overshoot, the socialist responds on a timescale much faster than a cell generation for both up- and downshifts in *σ*.

The consumer motif does not show any overshoot. It is relatively slow in up-shifts because it takes time to switch on the positive feedback. It is very fast in down shifts because the utilization system is already switched on in the initial state. However, if the downshift is so large that the positive feedback is switched off, then the response is relatively slow, on the timescale of one cell generation (see Fig. 1).

### Analysis of socialist dynamics

The socialist motif has two response times: the time required for *s* to reach within 95% of its final steady state value before (t_1_) and after (t_2_) the overshoot. Fig. 2 shows these quantities as a function of the perturbation size, Δ*σ*, for both up- and downshifts in *σ*. Fig. 2 also shows the time at which the overshoot reaches its maximum value.

The overshoot occurs because initially the concentration of the small molecule rises (for upshifts) or falls (for down-shifts) quickly; *σ* has changed at time zero, while *T* and *E* have not yet had time to change. Only after *E* starts being produced and *T* degraded, which occurs on the timescale of one cell generation, does the increase/decrease of *s* slow down. (For response times of *E* and *T*, see supplementary material, Fig. S3). This is enough time for the level of *s* to cross its final steady state level. Subsequently, the higher level of *s* means that *T* continues to fall, and *E* continues to rise, resulting in a decrease of *s* smoothly towards its final steady state level.

With up-shifts, Δ*σ*>0, Fig. 2 shows three interesting features: (i) the time to reach maximum of *s* is nearly constant, (ii) t_1_ decreases, but slower than linearly with Δ*σ*, and (iii) t_2_ increases as the log of Δ*σ*. The first observation shows that the quicker increase of *s* for larger perturbations is balanced by the higher level that *s* reaches before levelling off: The overshoot height *s_max_* is proportional to the perturbation size Δ*σ*∼*σ*.

The final steady state level of *s* rises with the size of the perturbation, but slower than linearly because of the overall negative feedback. However, the initial increase of *s* is proportional to Δ*σ*. Thus, for larger perturbations, the initial rise of *s* grows faster with the size of the perturbation than the increase in the final steady state. Accordingly t_1_ will decrease with Δ*σ* as indeed seen in Fig. 2. After the overshoot, the *s* level exponentially relaxes from its maximum to the final steady state level. The time required for such an exponential fall to 95% of the final level grows logarithmically with Δ*σ* as seen in Fig. 2. All of the above statements are explained in more detail in supplementary material, Text S1.

Similar observations can be made for the downshifts: (i) the time to reach maximum of *s* increases slowly, (ii) t_1_ increases, but slower than linearly with Δ*σ*, and (iii) t_2_ increases as the log of Δ*σ*. All of these follow from the fact that when there is a downshift perturbation, the level of *s* drops exponentially at a very high rate because *E* = 1 initially, so the consumption rate of *s* is at its maximum. Again, there is an overshoot because *E* and *T* change slowly on the timescale of one cell generation. For more details see supplementary material, Text S1.

Also in the supplementary material, (Fig S1 and Fig S2), we compare the behavior of the socialist with that of its individual loops. That is, we examine the effect of cutting one of the feedback loops in the original motif by keeping either *E* or *T* fixed at a constant level. Not surprisingly, the transport loop alone causes a larger overshoot, and constrains the final steady state more: at low values of *s*, which is the initial condition, the influx term is more dominant than the out-flux term, which is only proportional to *s*.

### Analysis of consumer dynamics

The response dynamics of the consumer motif differs qualitatively from that of the socialist motif. The initial effect of a jump in *σ* is a relatively slow build up of *s*, set by initial *T* and *E* that are near zero. Compared to the socialist, the initial transport is a factor 100 slower. Further, because of the positive feedback to transport there is no overshoot. This positive feedback counteracts overshoot because it pushes the final steady state up as much as *s* increases in the early stages of the response.

For up-shifts, as Δ*σ* is increased beyond the initial *σ* = 1, *s* reaches levels that trigger the positive feedback on transport: as *s* increases, *T* increases, which further speeds up the intake of *s*. Thus, the *s* vs. time curve bends upwards until the *s* level reaches very close to the final steady state value. As a result, the response time gradually decreases with Δ*σ* see Fig. 2. However, the timescale is always of the order of one cell generation. (See supplementary material, Text S1, for more details.)

For downshifts however, as long as Δ*σ* is below around 1000, the response is very fast. Essentially, in this regime, the system starts from an initial condition where both *E* and *T* are large, i.e. the system is “switched on”. For perturbations of size Δ*σ*<1000 the system remains in this switched on state: *E* and *T* lower a bit, but basically remain almost constant. When *E* and *T* are constant, and *σ* is changed, the *s* level relaxes exponentially to its new steady state at a rate *γ E*+1≈100 because *E* = 1. This is what makes the response time ≈1/100. However, when the perturbation is sufficiently large, then *E* and *T* also have to change substantially before the final steady state is reached. In that case, as usual, the timescale becomes around one cell generation. (For response times of *E* and *T*, see supplementary material, Fig. S4).

As with the socialist motif, we also examine the effect of reducing the original motif to a single loop by fixing the value of either *E* or *T* (Fig. S1 and S2.). Both individual loops reach their final steady state a little slower than the original motif: the transport loop alone is slower because the final steady state is much higher, while the metabolism loop alone is slower because of the overshoot. However, the overall response timescale remains around one cell generation.

### More complicated feedback regulation: iron homeostasis

In cellular metabolic systems the feedback associated to a given metabolite typically involves more that just two loops. For example in the case of the Galactose network of *E. coli*, there are in fact two core regulators that bind the small molecule D-galactose, and which each regulates transporters and enzymes [4], [5]. Similarly, several feedback loops are involved in the regulation of intracellular levels of the essential, but also poisonous iron ions, which is primarily governed by a socialist motif: Intracellular iron (*s*) binds to the Ferric uptake regulator, forming the active transcription factor complex FeFur (R*), which in turn activates the production of iron-using proteins (*E*) and also shuts down production of iron transporters (*T*). The socialist motif in iron regulation provides the negative feedback essential for maintaining homeostasis, i.e., keeping the steady state intracellular iron levels relatively insensitive to changes in the extracellular concentration.

In the socialist motif there are two response times, separated by a period of overshoot. Whether the first or the second response is important in the cell depends on whether the overshoot has a negative physiological effect. In the case of iron, it is likely that minimizing overshoot is crucial since intracellular iron is toxic at excessive quantities. Thus, it is necessary to supplement the core socialist motif in such a way that the overshoot is reduced while the homeostatic properties of the motif are retained.

**Figure 3:**
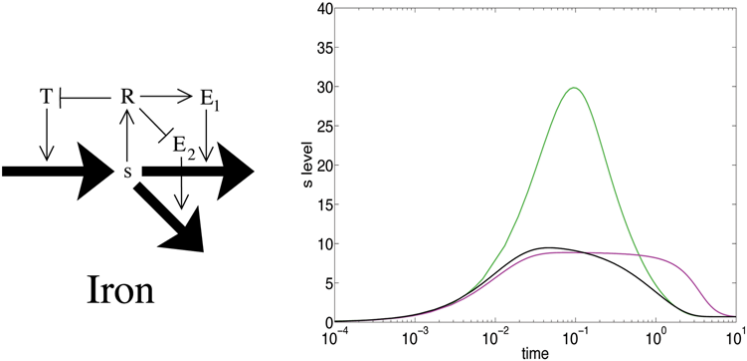
The iron homeostasis motif. The core motif in the iron regulation system is a socialist motif, enhanced by an extra positive feedback to metabolism. The plot shows a comparison between the *s* dynamics in the pure socialist motif (green line), a motif with a negative *T* feedback and positive *E* feedback (magenta line) and the combined iron motif (black line). The extra-cellular level is here changed from *σ* = 1 to 10^2^ and the motifs are tuned to reach the same final steady state level of *s* by altering the binding affinity of the regulator for *T* and *E*.

In E. coli there is in fact more than one channel for the consumption of iron. In addition to the incorporation of Fe into iron-using proteins, iron is also assembled into FeS clusters, thereby removing iron from the intracellular pool [14], [15]. This assembly can be mediated by two separate systems, Suf, and Isc [14], [15] The Suf system is part of a positive feedback loop which includes FeFur [12], [14]. As a result, the iron motif has (at least) three feedback loops as shown in Fig. 3. Fig. 3 also illustrates that this ‘iron motif’ has a substantially reduced overshoot. (See Fig. S5 for *s* dynamics for other Δ*σ*.)

The positive feedback loop involving Suf allows for an enzymatic conversion of *s* into another form (in this case FeS) before the normal channels of consumption have time to adjust. Thereby the build up of s, and the potentially damaging overshoot, is substantially reduced. We can conclude that in the iron response system, the socialist core motif is important in maintaining homeostasis with a near constant Fe level over large external ion concentrations. The positive metabolism loop involving Suf, on the other hand, plays an important role in reducing the overshoot. This is in agreement with studies of more detailed models of the iron response system [12].

## Discussion

Transport proteins and enzymes convert external resources to useful building blocks for the living cell. The regulation of these primary ‘workhorse’ proteins ensures that, 1) the cell optimizes its metabolism under different external conditions, and 2) the cell adjusts smoothly when the resources change. In the present paper we discussed how the dynamics of the change depended on the logic of the metabolite-regulation feedback loops. As metabolites are reshuffled on a very fast timescale, the response dynamics are mostly limited by the slowly changing protein concentrations. We find that a system with only negative feedback loops, like the socialist motif, always has a fast initial response with large ‘overshoot’ in metabolite concentration. The consumer motif on the other hand doesn't have overshoot but is typically quite slow to respond. The response is slow because it takes time to switch-on or switch-off the positive feedback to transport. If the perturbation is so small that the feedback isn't taken from a switched-on to a switched-off state, or vice versa, then the response is much faster. These are predictions that we hope will be tested in the future experimentally, by measuring dynamics of intracellular small molecule concentrations in living cells.

We note that for both motifs slow response basically means one cell generation. While this is relatively slow compared to the “fast” responses of 1/100 cell generation, it can actually be fast enough for practical purposes. Thus, a consumer motif that takes one cell generation to build up the level of a sugar molecule is adequately fast from a physiological point of view.

It is interesting that the deficiencies of the core motifs can be ameliorated by adding additional loops, while at the same time the benefits of the core motif are retained. In particular we found that positive feedback loops can be added to the socialist motif in such a way that the homeostatic property is largely preserved while dynamical aspects are substantially altered. This synergy of feedback loops is an important part of the functioning response of the iron response system in bacteria. The iron system represents a case study, and we would like to speculate that there is a general framework of rules for synergy of multiple combined feedback loops on the interface of a cell and its surrounding environment of small molecules.

## Supporting Information

Text S1 (0.19 MB PDF)

Figure S1 Response of the socialist and consumer motifs, and their individual loops, to up-shifts in σ. The left plots show the dynamics of intracellular s levels, for the corresponding motifs, for four different shifts in extracellular levels from σ = 1 to 1+Δσ with Δσ = 10, 100, 1000 and 10000. The two following columns show the s dynamics when only one of the loops are active, either the metabolic (E) or the transport (T) loop. When only the T loop is active E is kept small. When only the E loop is active, T is fixed, and kept small for the consumer motif and at its maximum for the socialist. (This is in correspondence with the levels of E and T for the initial conditions of σ = 1.)
(1.03 MB TIF)

Figure S2 Response of the socialist and consumer motifs, and their individual loops, to downshifts in σ. The left plots show the dynamics of intracellular s levels, for the corresponding motifs, for four different shifts in extracellular levels from σ = 10000 to 10000+Δσ with Δσ = −9000, −9900, −9990 and −9999. The two following columns show the s dynamics when only one of the loops are active, either the metabolic (E) or the transport (T) loop. When only the T loop is active E is kept at its maximum. When only the E loop is active, T is fixed, and kept small for the socialist motif and high for the consumer. (This is in correspondence with the levels of E and T for the initial conditions of σ = 10000.)
(1.00 MB TIF)

Figure S3 Response times for s (black), E (blue) and T (red) for the socialist motif. The left column displays the response time curves, when s is either an inhibitor of R (upper plot) or an activator (lower plot). The response time is defined as the time required to get to 95% of the final steady state levels of s. The response times are plotted as a function of the perturbation size for both up-shifts (left) and downshifts (right) in σ. For s, both the response time before and after the overshoot are plotted, with the shaded area in between marking the duration of the overshoot.
(0.96 MB TIF)

Figure S4 Response times for s (black), E and T (both red) for the consumer motif. The left column displays the response time curves, when s is either an inhibitor of R (upper plot) or an activator (lower plot). The response time is defined as the time required to get to 95% of the final steady state levels of s. The response times are plotted as a function of the perturbation size for both up-shifts (left) and downshifts (right) in σ.
(0.93 MB TIF)

Figure S5 The iron homeostasis motif. The core motif in the iron regulation system is a socialist motif, which is enhanced by an extra positive feedback to metabolism. These plots show the comparison between the s dynamics in the pure socialist motif (green line), a motif with a negative T feedback and positive E feedback (magenta line) and the combined iron motif (black line). The extra-cellular levels are here changed from σ = 1 to 10, 102 and 103 and the motifs are tuned to a fixed steady state level of s by altering the binding affinities of the regulator to E and T.
(0.88 MB TIF)
