# Supplementary material for: Dynamics of Uptake and Metabolism of Small Molecules in Cellular Response Systems
Source: PLoS One. 2009 Mar 17;4(3):e4923. doi: 10.1371/journal.pone.0004923 (PMC2654506; doi:10.1371/journal.pone.0004923)
Supplement: Text S1 — (0.19 MB PDF) [file pone.0004923.s001.pdf]

Supplementary material

Mathematical analysis of dynamics and response times

Socialist motif

With the socialist motif, for the up-shift perturbations we start $\sigma = 1$. In this steady state, T is large, while E and s are small: $T \approx 1$, $E \approx 0.2$, $s \approx 0.05$. Initially, just after the perturbation, when σ is changed to $\sigma' \equiv \sigma + \Delta\sigma$, E and T remain constant at their initial level. During this time, s evolves according to the equation

$$\frac{ds}{dt} = \sigma' T - (\gamma E + 1)s \approx \sigma' - 20s$$

$$\Rightarrow s(t) = s(0)e^{-20t} + \frac{\sigma'}{20}(1 - e^{-20t})$$

In other words, s starts from the initial value of $s(0) = 0.05$ and exponentially relaxes towards the target value of $s_f = \sigma'/20$ at a rate 20. Thus, the peak height of the overshoot is $\sigma'/20$ and the time taken to reach within 95 % of this level is $3/20 \approx 0.15$ (to get this we have assumed that the peak overshoot height is much larger than the starting level of s). Note that the overshoot height is proportional to the size of the perturbation, while the time is independent of it.

The final steady state level of s for the socialist motif grows slower than linearly with σ, i.e. $s(\infty) \sim (\sigma')^{\alpha}$ where $\alpha < 1$. The initial response time, t_1, can be calculated from the previously derived equation

$$\Rightarrow s(t) = s(0)e^{-20t} + \frac{\sigma'}{20}(1 - e^{-20t})$$

Essentially, for very small times only the first term on the right hand side is important

$$\Rightarrow e^{-20t} \approx s(t) / s(0)$$

$$\Rightarrow t_1 \sim 1 / \ln(\sigma')$$

i.e. t_1 decreases, but slower than linearly, as $\Delta\sigma$ is increased.

The second response time, t_2, is dominated by the time required for s to fall from the peak overshoot $\sigma'/20$ to the final $s(\infty) \sim (\sigma')^{\alpha}$, which is much smaller. Therefore, as a first approximation $t_2 \sim \ln(\sigma')$ (the larger the perturbation, the better this approximation). That is, t_2 grows as the logarithm of the perturbation size $\Delta\sigma$.

Overall, for the socialist motif subjected to up-shifts in σ, we have shown above that:
i) the peak overshoot height is $\sigma'/20$,
ii) the time of peak overshoot is independent of σ' and is ≈ 0.15,
iii) the initial response time decreases, slower than linearly, as $\Delta\sigma$ is increased,
iv) the second response time increases, slower than linearly, as $\Delta\sigma$ is increased.
This explains all the observations made in the main text about Figs. 1 and 2.

A similar analysis can be done for downshifts. We start with $\sigma = 10^4$, at which steady-state $s \approx 3$, $E \approx 1$, $T \approx 0.03$. Initially, just after the perturbation, when σ is changed to $\sigma' \equiv \sigma + \Delta\sigma$, E and T remain constant at their initial level. During this time, s evolves according to the equation

$$\frac{ds}{dt} = \sigma' T - (\gamma E + 1)s \approx 0.03\sigma' - 100s$$

$$\Rightarrow s(t) = s(0)e^{-100t} + 3\frac{\sigma'}{10^4}(1 - e^{-100t})$$

In other words, s starts from the initial value of s(0) = 3 and exponentially relaxes towards the target value of $s_f = 3\sigma'/10^4$ at a rate 100. Thus, the s level at peak overshoot is $3\sigma'/10^4$.

For sufficiently large perturbations ($\sigma \leq 1000$), this is much lower than the initial s level (the converse is true for up-shifts) the last term on the right hand side above is negligible, and the peak overshoot time ~const.- ln(σ). That is the peak overshoot time increases, slower than linearly, as the perturbation size Δσ is increased.

As for upshifts, the initial response time, t_1, can be calculated from the previously derived equation

$$\Rightarrow s(t) = 3e^{-100t} + 3\frac{\sigma'}{10^4}(1 - e^{-100t})$$

For very small times only the first term on the right hand side is important

$$\Rightarrow e^{-100t} = s(t)/r$$

$$\Rightarrow t_1 = 1/\ln(\sigma')$$

i.e. t_1 increases, but slower than linearly, as Δσ is increased.

The second response time, t_2, is dominated by the time required for s to increase from the peak overshoot $3\sigma'/10^4$ to the final steady state $s(\infty) \sim (\sigma')^{\alpha}$, which is much larger. For large enough perturbations, this is dominated by the timescale of one cell generation, which is required for E and T to reach their final steady states. Over this main timescale, t_2 also has contributions from t_1 and the peak overshoot time; therefore it also grows as Δσ is increased, but again slower than linearly.

Overall, for the socialist motif subjected to up-shifts in σ, we have shown above that:
i) the peak overshoot height is $3\sigma/10^4$,
ii) the time of peak overshoot grows slower than linearly as Δσ is increased,
iii) the initial response time increases, slower than linearly, as Δσ is increased,
iv) the second response time also increases, slower than linearly, as Δσ is increased.
This explains all the observations made in the main text about Figs. 1 and 2.

Consumer motif

The analysis of the consumer motif is easier. For upshifts, we start with $\sigma = 1$, and $s \approx 0.005$, $T = E \approx 0.01$. In this case the positive feedback is off. As soon as $\Delta\sigma$ is larger than around 1, the perturbation is big enough to start switching on the positive feedback. Already for $\Delta\sigma = 10$ the positive feedback is almost completely on. To get to this on state both E and T must increase, which happens on a timescale of one cell generation. Thus, the response of the consumer to up-shifts is of that timescale.

For downshifts, we start with $\sigma = 10^4$. At this value, $s \approx 100$, $T = E \approx 1$, i.e. the positive feedback is switched on. $\Delta\sigma$ has to be quite large (roughly > 9990) for the perturbation to be large enough to start switching the feedback off. For $\Delta\sigma < 9990$ the feedback remains on, with $T = E \approx 1$. In this regime, the s dynamics is given by

$$\frac{ds}{dt} = \sigma' - 100s$$
$$\Rightarrow s(t) = s(0)e^{-100t} + 0.01\sigma'(1 - e^{-100t})$$

Thus, for small enough perturbations the response time is $\approx 3/100 = 0.03$. For larger perturbations, where the feedback is switched off, and E and T change significantly, the response time as usual becomes of the order of one cell generation.

Thus, as seen in Figs. 1 and 2 of the main text,
i) for up-shifts the consumer motifs responds on the timescale of one cell generation,
ii) for small enough down-shifts the response time is of the order of one hundredth of a cell generation,
iii) for very large down-shifts, the response is again of the order of one cell generation.
