# Supplementary figures and images for: Dynamics of Uptake and Metabolism of Small Molecules in Cellular Response Systems
Source: PLoS One. 2009 Mar 17;4(3):e4923. doi: 10.1371/journal.pone.0004923 (PMC2654506; doi:10.1371/journal.pone.0004923)

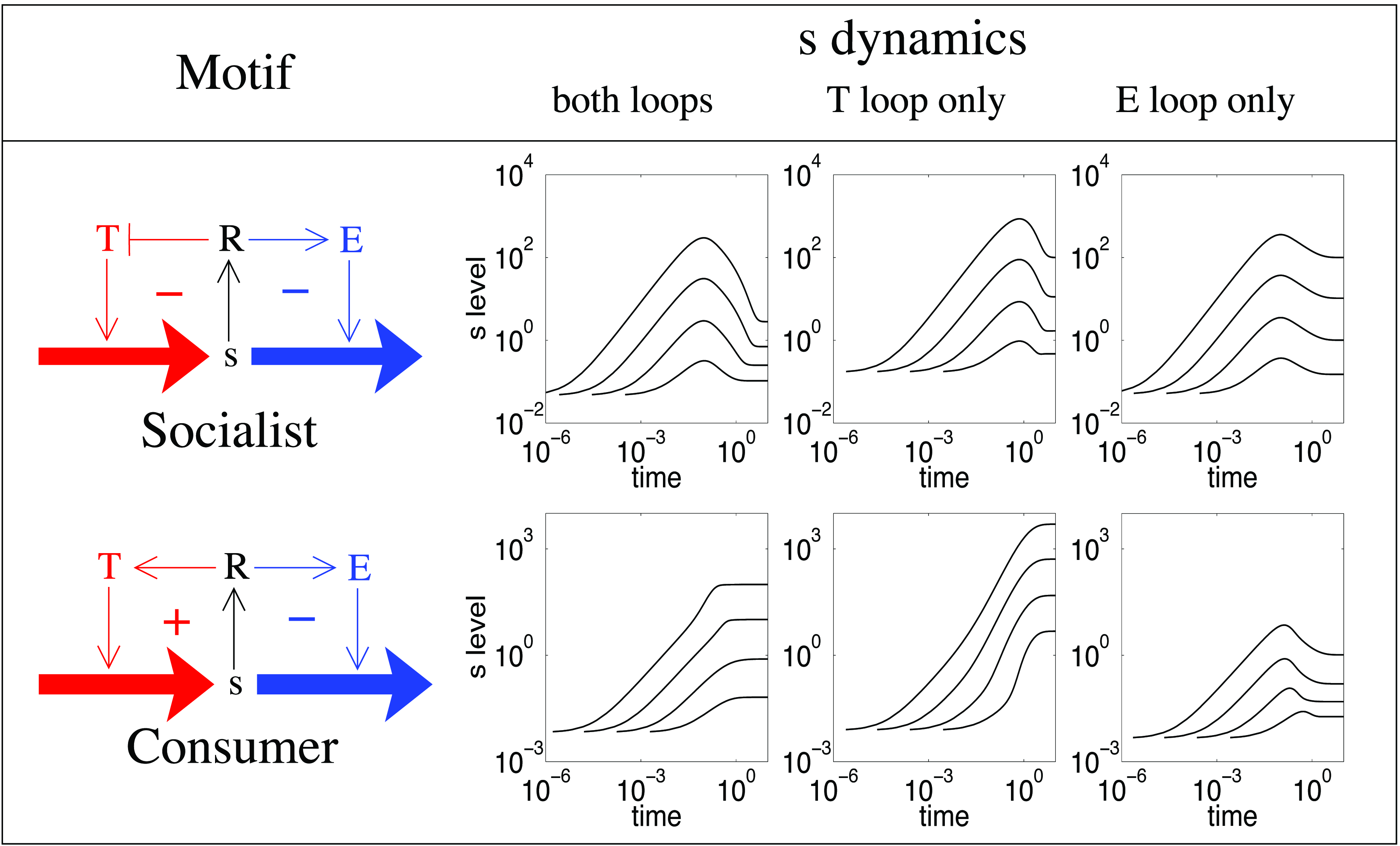

Supplement: Figure S1 — Response of the socialist and consumer motifs, and their individual loops, to up-shifts in σ. The left plots show the dynamics of intracellular s levels, for the corresponding motifs, for four different shifts in extracellular levels from σ = 1 to 1+Δσ with Δσ = 10, 100, 1000 and 10000. The two following columns show the s dynamics when only one of the loops are active, either the metabolic (E) or the transport (T) loop. When only the T loop is active E is kept small. When only the E loop is active, T is fixed, and kept small for the consumer motif and at its maximum for the socialist. (This is in correspondence with the levels of E and T for the initial conditions of σ = 1.) (1.03 MB TIF) [file pone.0004923.s002.tif]

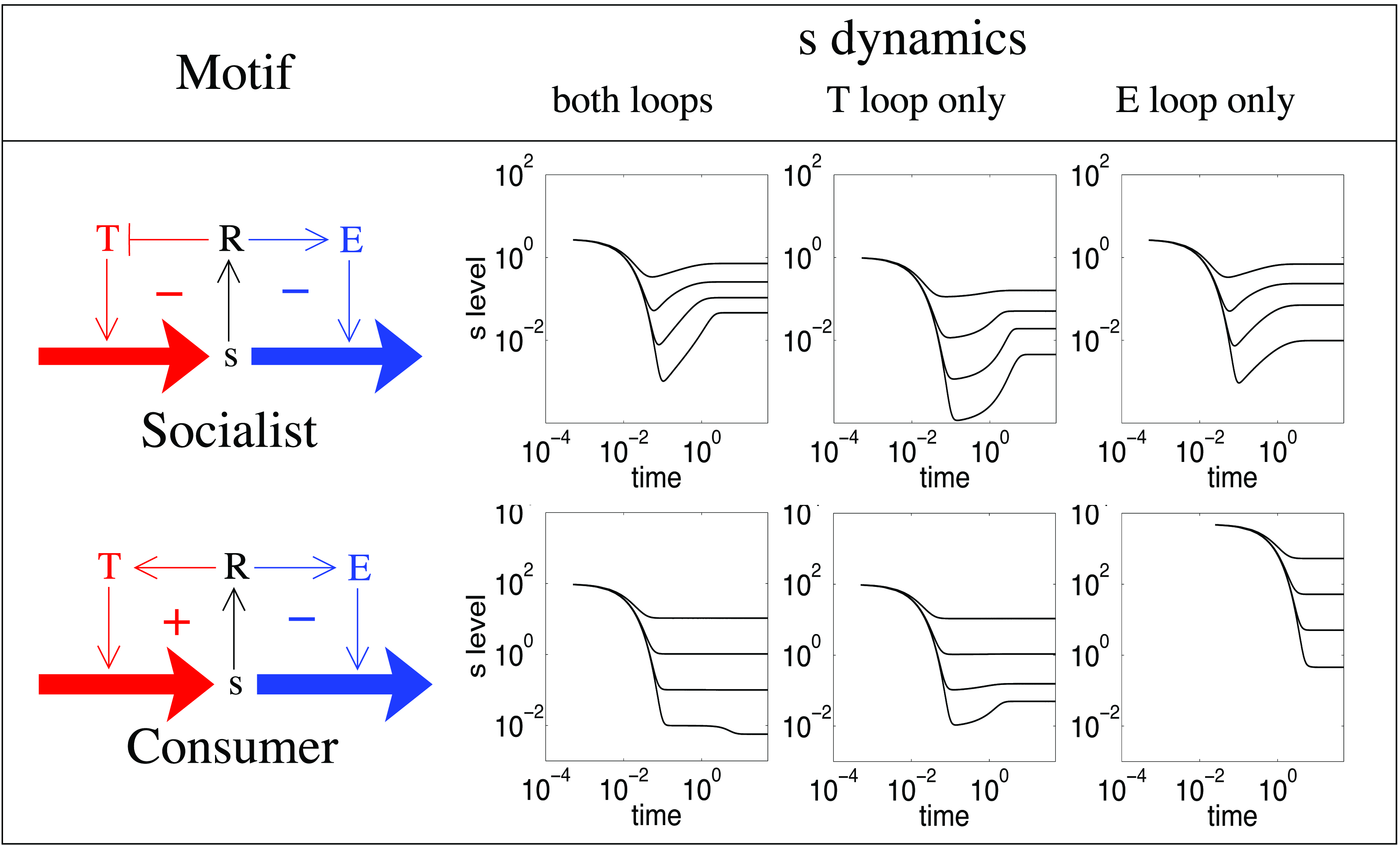

Supplement: Figure S2 — Response of the socialist and consumer motifs, and their individual loops, to downshifts in σ. The left plots show the dynamics of intracellular s levels, for the corresponding motifs, for four different shifts in extracellular levels from σ = 10000 to 10000+Δσ with Δσ = −9000, −9900, −9990 and −9999. The two following columns show the s dynamics when only one of the loops are active, either the metabolic (E) or the transport (T) loop. When only the T loop is active E is kept at its maximum. When only the E loop is active, T is fixed, and kept small for the socialist motif and high for the consumer. (This is in correspondence with the levels of E and T for the initial conditions of σ = 10000.) (1.00 MB TIF) [file pone.0004923.s003.tif]

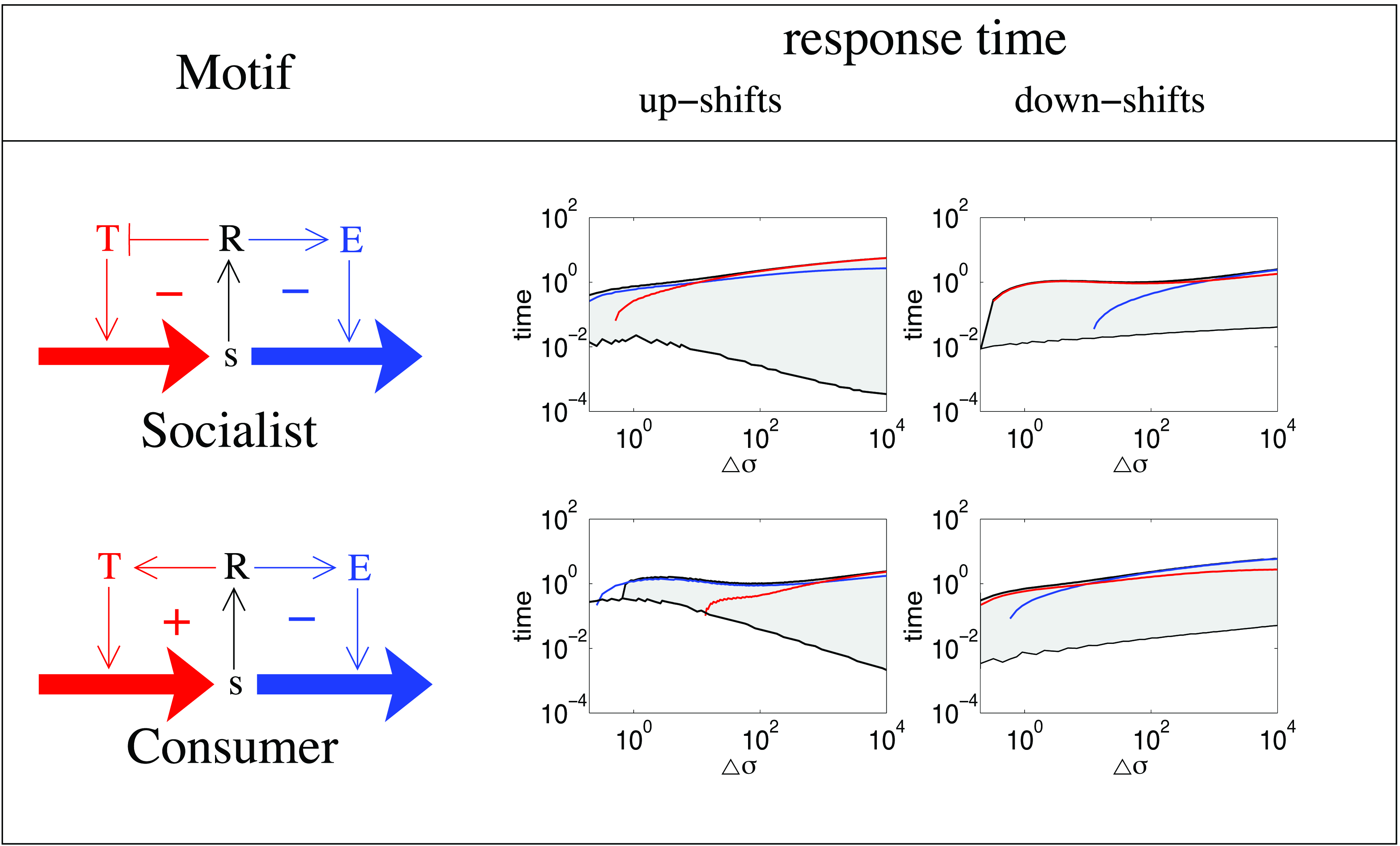

Supplement: Figure S3 — Response times for s (black), E (blue) and T (red) for the socialist motif. The left column displays the response time curves, when s is either an inhibitor of R (upper plot) or an activator (lower plot). The response time is defined as the time required to get to 95% of the final steady state levels of s. The response times are plotted as a function of the perturbation size for both up-shifts (left) and downshifts (right) in σ. For s, both the response time before and after the overshoot are plotted, with the shaded area in between marking the duration of the overshoot. (0.96 MB TIF) [file pone.0004923.s004.tif]

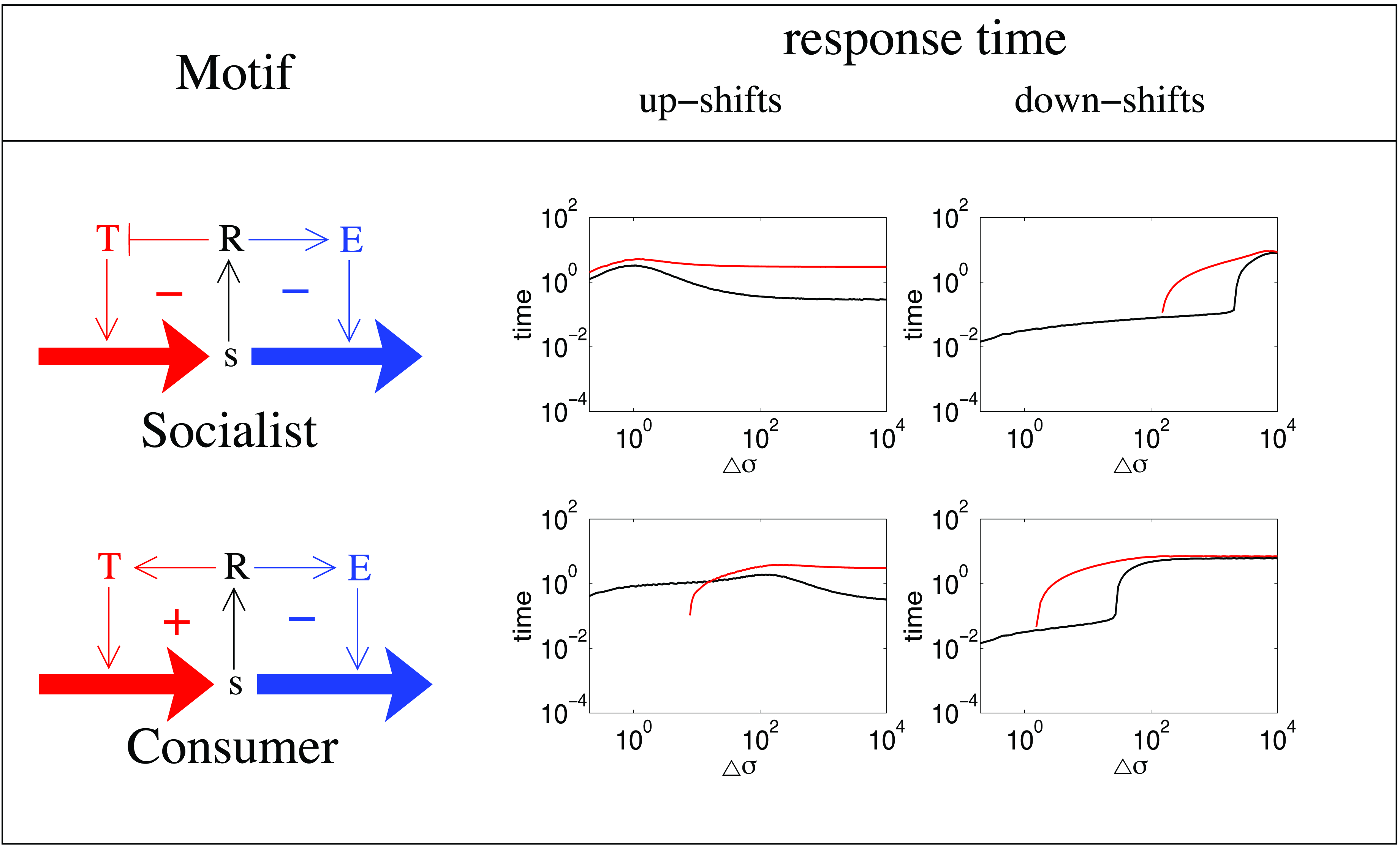

Supplement: Figure S4 — Response times for s (black), E and T (both red) for the consumer motif. The left column displays the response time curves, when s is either an inhibitor of R (upper plot) or an activator (lower plot). The response time is defined as the time required to get to 95% of the final steady state levels of s. The response times are plotted as a function of the perturbation size for both up-shifts (left) and downshifts (right) in σ. (0.93 MB TIF) [file pone.0004923.s005.tif]

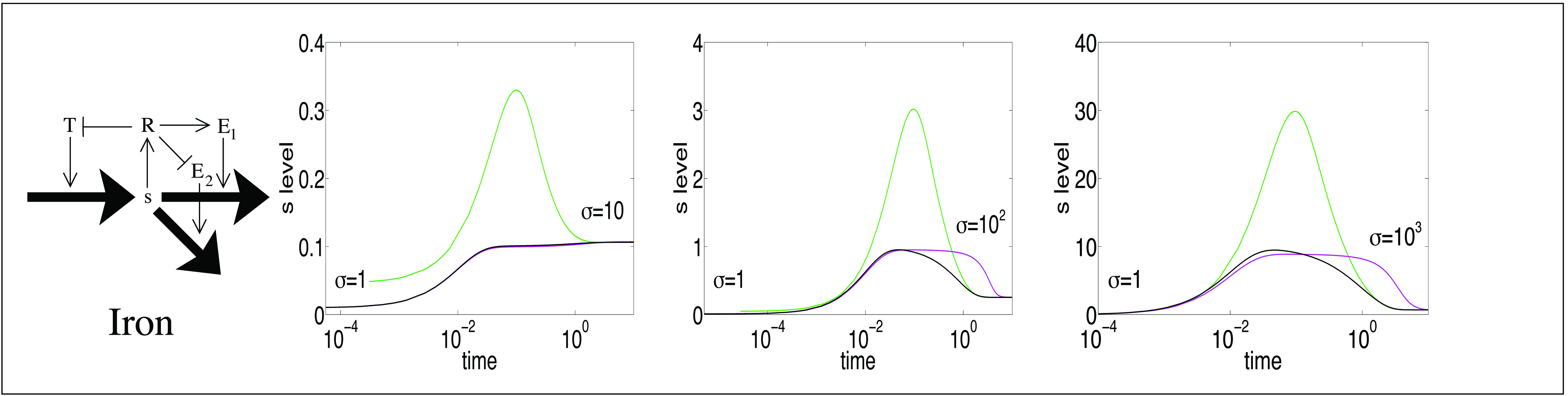

Supplement: Figure S5 — The iron homeostasis motif. The core motif in the iron regulation system is a socialist motif, which is enhanced by an extra positive feedback to metabolism. These plots show the comparison between the s dynamics in the pure socialist motif (green line), a motif with a negative T feedback and positive E feedback (magenta line) and the combined iron motif (black line). The extra-cellular levels are here changed from σ = 1 to 10, 102 and 103 and the motifs are tuned to a fixed steady state level of s by altering the binding affinities of the regulator to E and T. (0.88 MB TIF) [file pone.0004923.s006.tif]
